# Evasion of Plant Innate Defense Response by *Salmonella* on Lettuce

**DOI:** 10.3389/fmicb.2020.00500

**Published:** 2020-04-03

**Authors:** Nicholas Johnson, Pushpinder K. Litt, Kalmia E. Kniel, Harsh Bais

**Affiliations:** ^1^Department of Plant and Soil Sciences, University of Delaware, Newark, DE, United States; ^2^Delaware Biotechnology Institute, University of Delaware, Newark, DE, United States; ^3^Department of Animal and Food Sciences, University of Delaware, Newark, DE, United States

**Keywords:** *S. enterica*, *L. monocytogenes*, T3SS, stomata, innate defense response, food safety, pattern triggered immunity, hormonal response

## Abstract

To establish host association, the innate immune system, which is one of the first lines of defense against infectious disease, must be circumvented. Plants encounter enteric foodborne bacterial pathogens under both pre- and post-harvest conditions. Human enteric foodborne pathogens can use plants as temporary hosts. This unique interaction may result in recalls and illness outbreaks associated with raw agricultural commodities. The purpose of this study was to determine if *Salmonella enterica* Typhimurium applied to lettuce leaves can suppress the innate stomatal defense in lettuce and utilization of UD1022 as a biocontrol against this ingression. Lettuce leaves were spot inoculated with *S.* Typhimurium wild type and its mutants. Bacterial culture and confocal microscopy analysis of stomatal apertures were used to support findings of differences in *S.* Typhimurium mutants compared to wild type. The persistence and internalization of these strains on lettuce was compared over a 7-day trial. *S.* Typhimurium may bypass the innate stomatal closure defense response in lettuce. Interestingly, a few key T3SS components in *S.* Typhimurium were involved in overriding stomatal defense response in lettuce for ingression. We also show that the T3SS in *S.* Typhimurium plays a critical role in persistence of *S.* Typhimurium *in planta*. *Salmonella* populations were significantly reduced in all UD1022 groups by day 7 with the exception of *fljB* and *invA* mutants. *Salmonella* internalization was not detected in plants after UD1022 treatment and had significantly higher stomatal closure rates (aperture width = 2.34 μm) by day 1 compared to controls (8.5 μm). *S.* Typhimurium SPI1 and SPI2 mutants showed inability to reopen stomates in lettuce suggesting the involvement of key T3SS components in suppression of innate response in plants. These findings impact issues of contamination related to plant performance and innate defense responses for plants.

## Introduction

*Salmonella* remains a critical foodborne pathogen given its ability to persist in various environments and hosts (plant, animal, human). Microbial contamination can originate from countless areas along the farm to fork continuum. At greatest risk are those aspects of contamination that can occur within the pre-harvest environment, whereby microbial contamination can come in contact with plant foliar tissues from water, soil amendments, wind, birds, insects, animals, and other fomites including food contact surfaces on-farm and at packaging facilities (Beuchat, 2002; Brandl, 2006; Berger et al., 2010). Risk of contamination of plants in the field likely occurs *via* direct and indirect mechanisms (Monaghan and Hutchison, 2012). Laboratory studies suggest that bacterial pathogens on plants in the field decrease quickly over the first 3 days, but low numbers continue to persist for several weeks, which may cause human health issues (Erickson, 2012). This study explores potential host-microbe ingression between plants and plant associated bacteria, which may be involved in plant contamination (Schikora et al., 2008; Kumar et al., 2012; Hsu and Micallef, 2017).

The Centers for Disease Control and Prevention (CDC) attributed 46% of illnesses to fresh produce and particularly leafy greens (Painter et al., 2013). Indicating that greater and more sophisticated efforts are needed to prevent contamination on these commodities that cause foodborne illness. Plants grow in close association with large communities of microbes, yet comparatively little is known about the diversity of microbes that associate with plants, and their interactions and effects on performance, crop yields and plant protection. The fields of plant science and food microbiology have been merging over the past few years in the best interest of produce safety, however, critical data gaps remain. Importantly, there is limited knowledge about the modes of entry human bacterial pathogens may utilize for plant ingression, which may occur through several routes, including openings in roots, on the cortex, or epidermis (Deering et al., 2012; Erickson, 2012; Hirneisen et al., 2012).

Reports have shown that human pathogens that infect mammals and other higher animals are able to infect plants (Bishop and Davis, 1990; Plotnikova et al., 2000; Rahme et al., 2000). Studies using *Pseudomonas aeruginosa* showed that few human pathogens use conserved virulence factors to infect multiple hosts including plants (Rahme et al., 2000). Like *P. aeruginosa*, *Salmonella enterica* serovar Typhimurium also uses plants as alternative hosts to humans and other animals. It is shown that these bacteria adhere to plant surfaces and actively infect the interior of plants. Using *Arabidopsis thaliana* as a model system, it was shown that, *S*. *enterica* serovar Typhimurium suppresses plant defense responses mediated by a type III secretion mechanism (Schikora et al., 2008). In addition, it was also shown that several *S. enterica* serovars exhibit variations in pathogenicity, on different plant species revealing different innate defense response toward these bacteria (Schikora et al., 2011, 2012). An important feature of *Salmonella* infections in plants is its ability to adhere to plant surface. Interestingly, it was shown that several *S. enterica* serovars adhere to plant surfaces better then pathogenic *E. coli* strain O157:H7 (Barak et al., 2002). A mutant screen identified over 20 mutants impaired in surface attachment in *S. enterica* serovars on *Medicago sativa* (alfalfa) sprouts (Barak et al., 2005). Majority of genes involved in attachment of *Salmonella* to alfalfa were related to surface-exposed aggregative fimbria nucleator curli (*agfB*) and for the global stress regulator *rpoS* which regulates the production of curli, cellulose and other adhesins (Barak et al., 2005). It is shown that the involvement of biofilm operons and swarming genes modulate attachment to plant surface, in the phyllosphere and light sensing leading to modification in stomatal aperture (Barak et al., 2009; Kroupitski et al., 2009). Contrastingly, not much is known about how *Salmonella* spp. infect and ingress in leafy green plants. In mammalian systems, *S. enterica* serovar Typhimurium uses both pathogenicity islands 1 and 2 (SPI1, SPI2) to cause virulence (Coburn et al., 2007). Primary function of the SPI1 is cellular invasion, while the SPI2 is required for cellular survival and reproduction in the *Salmonella* containing vacuole (SCV) (Ohl and Miller, 2001; Löber et al., 2006). SPI1 virulence island is controlled via several genes; *hilC*, *hilD*, and *sirA*/*barA* that have been identified as regulators of *hilA* and downstream products in the SPI1 (Ellermeier et al., 2005). Regulation of *hilA* controls all SPI1 functions, including the T3SS. It is thus evident that the genetic equipment of *Salmonella*, previously thought to be animal-infection specific, plays an important role in the infection of animals and plants alike (Ellermeier et al., 2005). Within SPI2 SseB is a chaperone protein required for functional T3SS, mutations effect phase two pathogenicity that occurs within the *Salmonella* containing vacuole within mammalian cells (Ellermeier et al., 2005).

Foodborne pathogens can contaminate at any part of the farm to fork continuum, most often it occurs during preharvest through non-specific sources or irrigation water. A recent multistate outbreak in the United States was linked to a contaminated water source on a farm in California, and no farm is immune to accidental contamination via feces from wildlife or presence of native soil pathogens such as *Listeria* spp. (CDC, 2018, 2012). It is known that various beneficial microbes induce defense response in plants (Rosier et al., 2018). It was shown that root association of a beneficial microbe, *Bacillus subtilis* strain UD1022 (hereafter UD1022) protects *Arabidopsis* plants from aerial pathogens by modulating stomatal apertures (Kumar et al., 2012). The stomatal aperture reduction by UD1022 didn’t modify the gaseous exchange process in plants and was transient in nature (Kumar et al., 2012). The stomatal closure by UD1022 in *Arabidopsis* was dependent on ABA and SA pathways (Kumar et al., 2012). We also showed that the reduction of stomatal aperture by UD1022 may be translated to leafy greens such as lettuce and spinach plants (Markland et al., 2015).

Here, we developed a model patho-system with lettuce as a plant system to evaluate the ingression and persistence of *S. enterica* serovar Typhimurium 14028 (henceforth *S.* Typhimurium) *in planta*. We observed that *S.* Typhimurium may bypass the early stomatal closure defense response in lettuce. Our observation shows that *S.* Typhimurium subverts the immune system and prevents stomatal closure at times when it normally would be closed as part of the innate immune response. Interestingly, few key T3SS components in *S.* Typhimurium were involved in overriding stomatal defense response in lettuce for ingression. We also show that the T3SS in *S.* Typhimurium plays a critical role in persistence of *S.* Typhimurium *in planta*. Gene expression analysis shows that *S.* Typhimurium may perturb the ABA biosynthesis pathway to subvert stomatal defense. We also showed that root application of a UD1022 reduces stomatal aperture leading to reduced ingression by *S.* Typhimurium. Interestingly, co-inoculation of UD1022 with *S.* Typhimurium overrides ability of *S.* Typhimurium to reopen stomates in lettuce. Our findings show a development of patho-system involving leafy green specie with *S.* Typhimurium. The model system provides many possibilities of understanding molecular, biochemical and physiological networks that underpin this unique plant-human pathogen interaction.

## Materials and Methods

### Plant Growth Methods

*Lactuca sativa* (Family: Asteraceae) var. Black Seeded Simpson was purchased from Johnny’s Select Seed. Before cultivation all seeds were stratified for 48 h on a damp paper towel in a conical tube at 4°C, the seeds were then soaked in 50% bleach for 8 min in the same 50 mL conical tube before being washed thrice with a minimum of 25 mL sterile water each time. This cleaning method resulted in only one plant in 2 years that had a mold presence and no loss of seed viability or germination rate. The clean seeds were placed on MS agar with 1% sucrose and grown under a 1750 (PAR = 200–230) lumen grow light grid at room temperature (25 ± 3°C) for 2 weeks with a 12 h photoperiod or placed in sterilized magenta boxes with autoclaved hydroponic clay and a modified Sonneveld Solution (Mattson and Peters, 2014). For persistence internalization assays: *Lactuca sativa* var. Black Seeded Simpson was planted in a sterile pro-mix made up of 85% Canadian sphagnum peat moss with perlite, vermiculite, dolomitic and calcitic lime, a wetting agent, and mycorrhizae (Premier Tech Horticulture, Quakertown, PA, United States) in a seed tray with holes at the bottom (4 cm × 3.5 cm × 4.5 cm in dimension; T.O. Plastics, ON, Canada) and placed in another plastic container. Trays were maintained in Biosafety Level 2 growth chamber (Percival Scientific, Boon, IA, United States) at 20°C with 12 h photoperiod and at a constant relative humidity of 60% (Markland et al., 2015). Plants were irrigated by pouring water into the bottom plastic container to saturation of the soil.

### Bacterial Culture Preparation

All bacterial strains were kept at −80°C freezer in 20% glycerol for long term storage. Prior to use each bacterial strain was streaked onto a complex-media (Tryptic Soy or Luria-Bertani) containing necessary antibiotics where applicable (Supplementary Table S1). Each plate was incubated for 16–24 h at 30°C, and re-streaked from the glycerol stocks as needed. Before experimental use, a single colony from solid media was moved into liquid media via sterile loop technique and incubated at 30°C overnight on an orbital shaker at 200 rpm. Following incubation, liquid bacterial cultures were aliquoted into conical tubes and centrifuged for 15 min at 4000 rpm and washed twice with 25 mL PBS buffered to a pH of 7.4, followed by a final suspension in PBS. The optical density was measured at 600 nm with a Bio-Rad SmartSpec + spectrophotometer (Bio-Rad Inc.) and adjusted to the working concentration of 10^7^ CFU mL^–1^ in sterile DI water. For all experiments utilizing live cells, bacterial cultures were prepared in the manner as described above.

### Bacterial Culture Filtrate (CFL) Preparation

*Salmonella* Typhimurium, *sseB*, or *hilD* were grown in 50 mL M9 media with 2% dextrose for 24 h at 30°C, the resulting suspension was measured with a spectrophotometer to check for similar cell density. The culture was then centrifuged for 15 min at 4000 rpm and filter sterilized through a 0.22 μm filter. Heat treated CFL was prepared in an identical fashion followed by 3 h incubation in a 65°C water bath before use. Contamination was checked for via plating 100 μL onto LB agar; no contamination was ever observed.

### Stomatal Assay

Light adapted 2-week-old lettuce plants grown on MS agar were brushed with sterilized water, a suspension of 10^7^ cfu mL^–1^ of the bacteria listed in Supplementary Table S1, various MAMPs [S. *typhimurium* LPS 10 μg mL^–1^, *Pseudomonas aeruginosa* LPS 10 μg mL^–1^, FLG22 peptide at 10 μg mL^–1^], or plant growth hormones [5 μM SA, or 20 μM ABA]. Co-inoculation of bacteria and a plant growth hormone [5 μM SA, or 20 μM ABA] occurred by brushing the plants (Markland et al., 2015) first with ABA or SA followed by bacteria with a separate sterilized brush to avoid cross contamination. Following inoculation, the plants were incubated at room temperature under the previously described grow light grid for 3–12 h, stomatal aperture were monitored at 3 h post inoculation or 3, 6, and 12 h post inoculation (Markland et al., 2015). For image analysis inoculated leaves were excised with alcohol sterilized forceps. From the excised leaf a small circle was removed with a potato corer which was stained with propidium iodide (PI) for 8 min followed by a light rinse with deionized water and placed abaxial side up under a glass block in chambered cover glass (NUNC/VWR). Directly after staining plant samples were imaged with a Zeiss 710 Confocal Microscope.

### *B. subtilis* (UD1022) Treatment for Modulation of Stomatal Apertures

A suspension of *B. subtilis* strain UD1022 (henceforth UD1022) was made as previously described, final suspension in sterile nanowater water was to a concentration of 10^5^ CFU mL^–1^. Lettuce grown in magenta boxes received 10 mL of this dilute culture for a total of 10^6^ cells per magenta box. The boxes were then returned to the growth chamber for a 48 h incubation with a 24 h photoperiod after the first 24 h. Following light adaptation *S.* Typhimurium WT, *hilD, sseB*, or water inoculations (control) were completed as described above after the 48 h incubation and imaged at 3 and 6 h post inoculation. Alternatively, UD1022 treated plants were taken for imaging directly after UD1022 inoculation, with the remaining plants placed in the growth chamber. Samples were imaged at 0, 4, 8, 24, 36, 48, and 60 h post inoculation. At identical time points using different plants a calibrated porometer was utilized to gather 3–4 measurements from three plants per time point.

### Confocal Imaging Parameters

Imaging was performed using a Zeiss710 Inverted Laser Scanning Microscope located in the University of Delaware Bioimaging Core, samples were imaged at 0, 3, 6, and 12 h post inoculation. PI stain was excited with a 561 nm laser through a 488/561 bandpass filter with the emission spectra set to 580–640 nm. Images were captured with 2048 × 2048 pixels per frame and 20× magnification +1× digital zoom for 425.1 μm per frame. Settings were consistent over all samples with exception to, digital gain, and aperture width. In addition to PI fluorescent bands, the phase image in grayscale was retrieved for each micrograph.

### Cryo-SEM Imaging

Light adapted 2-week-old lettuce was brushed with a suspension of either *S.* Typhimurium WT, *hilD*, *sseB*, or left un inoculated. To prepare leaves for SEM small holes were removed from each sample leaf with a potato corer (1 per leaf) to retrieve symmetrical circles for imaging, each leaf circle was placed adaxial side up on a gold block with tissue mounting fluid and carbon-black. After mounting, the leaves were flash frozen by being plunged into liquid nitrogen and contained under a vacuum, the block was then transferred to the cryo-SEM chamber and brought to −120°C, the leaves were then sublimated at −90°C to remove ice-films on the surface, and finally sputter coated with gold and palladium before imaging took place at −120°C. Leaves treated with *S.* Typhimurium WT, *sseB*, *hilD* were imaged at 3 and 6 h post inoculation, with a references plant sample left uninoculated (0 h post inoculation). Each leaf subsample was imaged at a magnification of 500X. All images were processed in the same fashion as those from confocal based leaf assays, albeit with an adjusted scale.

### Gene Expression Studies

Light adapted 2-week-old lettuce was brushed with either water or *S.* Typhimurium in the identical fashion to the above stomatal assay. At 0 h post inoculation, 3 h post inoculation, and 6 h post inoculation samples were flash frozen in liquid nitrogen followed by RNA extraction following the Qiagen RNeasy protocol. In order to quantify the expression levels within *S.* Typhimurium treated lettuce the RNA was converted to complementary DNA via reverse transcription PCR. Rt-PCR was completed with Multiscribe^®^ Reverse Transcriptase (Thermo-Fischer), for each reaction 1000 ng of RNA was used. The master mix was made as follows; 2 μL RT-Buffer, 2 μL RT-random Primers, 0.8 μL dNTP-Nucleotides, 1 μL Multiscribe Reverse Transcriptase, 1000 ng RNA, and enough nuclease free water to bring the reaction volume to 20 μl. PCR parameters were as follows for every sample: 25°C for 10 min, 37°C for 2 h, 85°C for 5 min, and 4°C until samples were moved to a −20°C freezer for long term storage. A semi-quantitative PCR technique using a DreamTaq Green Polymerase and protocol (Thermofischer) was employed to check cDNA viability and relative expression of genes. The primers and PCR conditions used can be found in Supplementary Tables 2A,B, 3. Following PCR gels were imaged using a Biorad Gel Dock, individual bands were measured using Image J and data added to Microsoft Excel. Relative expression of each primer was averaged and normalized against Actin. Significant differences were found using a Student’s *t*-test at a significance level of *p* < 0.05.

### Persistence and Internalization of *S.* Typhimurium Mutants Lacking Type-III Secretion System (T3SS) Virulence Factors and Flagellar Genes in Lettuce Grown Under Greenhouse Conditions

Two-week-old lettuce plant leaves were spot-inoculated randomly with 120 μl (6 droplets) of respective *Salmonella* culture on the leaf surface. Inoculated leaves were placed in the growth chamber for 2 h, to facilitate bacterial attachment. Leaf samples were collected for each treatment group separately and processed to enumerate surviving *Salmonella* populations on day 0, 1, 3, 5, and 7. A sample of 6 plants (total of 12 leaves in each) was collected on each sampling day and split into two equal sections for bacterial enumeration and pathogen internalization assay. To enumerate, leaf samples (6 leaves from plant) were weighed in individual Whirl-Pak^TM^ bags (Nasco, Fort Atkison, WI, United States) and submerged in 0.1% buffered peptone water (BPW; Oxoid Ltd., Basingstoke, Hampshire, England) in a 1:9 ratio. The sample was mixed for 2 min at 230 rpm in a stomacher (BagMixer^®^ 400 S, Interscience), the resulting mix was serially diluted in 0.1% BPW and plated on TSA with antibiotic (Supplementary Table S2), or by a mini-MPN method (Sharma et al., 2016). *Salmonella* colonies were counted after 22–24 h of incubation at 37°C.

### *Bacillus subtilis* UD1022 Colonization of *S.* Typhimurium Mutants Lacking Flagellar and Type-III Secretion System (T3SS) Virulence Factors

Roots of 14 days old plant were inoculated with live culture of *B. subtilis* UD1022 (∼10^8^ CFU/ml) by adding culture to irrigation water to a saturation. Plants for maintained in bio-chamber for 48 h at 20°C with a 12 h photoperiod and at a constant relative humidity. After 48 h, lettuce leaves were spot inoculated randomly with respective *Salmonella* mutant culture on the leaf surface and leave samples were processed as explained above. Treatment groups also included, negative control, *B. subtilis* UD1022 only control.

### Internalization Assay

To detect pathogen internalization in lettuce, lettuce leaves were spot inoculated with 120 μl (6 droplets) of respective *Salmonella* culture on the leaf surface. Inoculated leaves were placed in the growth chamber for 2 h, to facilitate bacterial attachment. Leave samples were collected on day 0, 1, 3, 5, and 7. Sampled leaves were surface disinfected by immersing them in 80% ethanol for 10 s, followed by 10 min dip in 0.1% mercuric chloride (Markland et al., 2015 and Erickson et al., 2010). Leaves were washed with 10 mL sterile water and grounded using a rubber mallet. Subsequently, leaves were transferred to a fresh Whirl-Pak bags and re-suspended in 0.1% BPW. Re-suspended leaves were homogenized for 2 min at 230 rpm in a stomacher and 1 mL of the resulting mix was transferred to 9 mL TSB. Inoculated TSB was incubated for 18–24 h at 37°C. After incubation, a loop full sample from each tube was streaked onto TSA plates and incubated for 18 h at 37°C. Plates were observed for *Salmonella* colonies.

### Statistical Analysis

All the experiments were repeated in triplicate per treatment. Bacterial populations, recovered at each sampling point, were converted to log10 CFU/g or MPN/g, and the mean values of the three replicates were obtained. The limit of detection was 1 log CFU/g. Stomatal width at each time point was captured with confocal microscopy and measured using ImageJ. Data were analyzed using a one-way ANOVA to determine the effect of treatments, and student’s *t*-test was performed to compare the means of bacterial populations, stomatal width, and relative gene expression over time by using JMP software (JMP v.14; SAS Institute Inc., Cary, NC) at a significance level of *P* < 0.05.

## Results

### *S.* Typhimurium Treatment Modifies Stomatal Aperture in Lettuce

**FIGURE 1:**
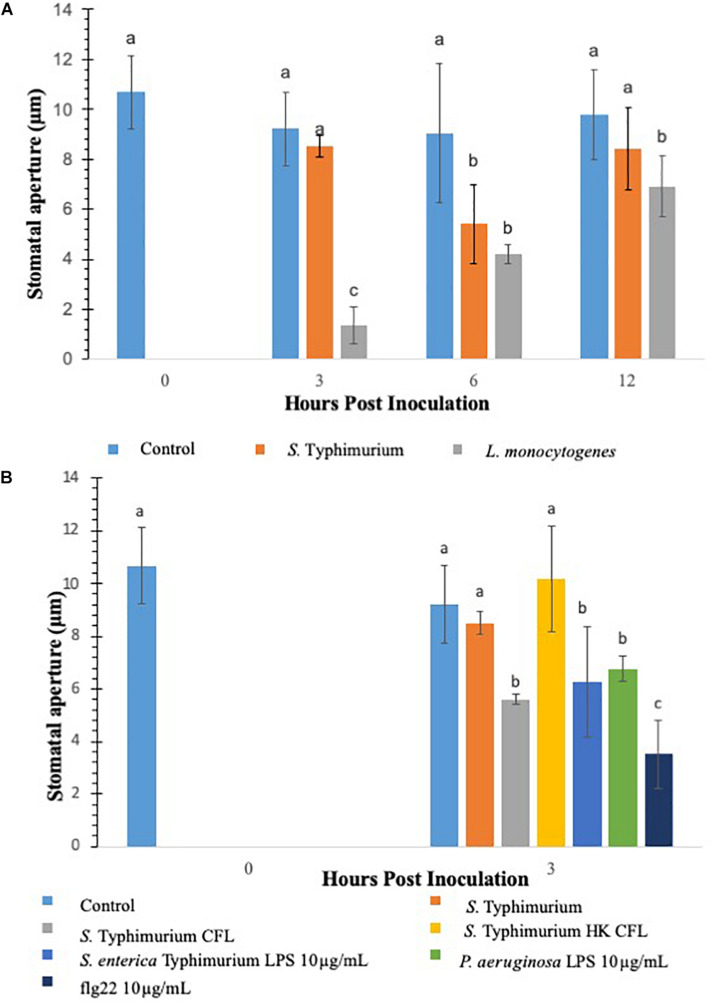
Comparison of innate immune response in terms of stomatal closure by *L. monocytogenes* and *S. enterica* Typhimurium on lettuce. Aperture changes at 0 h remains unchanged, and 0 h represents only the water control. Different letters signify a difference at *p* < 0.05. For each time point the average was found from three plants with *n* = 40–60 stomata imaged per plant **(A)**. Comparison of innate immune response in terms of stomatal closure by *S. enterica* Typhimurium and MAMPs on lettuce **(B)**. Aperture changes at 0 h remains unchanged, and 0 h represents only the water control. Different letters signify a difference at *p* < 0.05. For each time point the average was found from three plants with *n* = 40–60 stomata imaged per plant.

Previous studies have shown that stomata act as an entry points for various plant and human pathogens to ingress in plants (Kumar et al., 2012; Markland et al., 2015). To determine if different opportunistic pathogens modulate stomatal aperture in lettuce, in this study lettuce leaves were (2-weeks-old) inoculated with *S.* Typhimurium or *L. monocytogenes*. This study evaluated two methods to measure stomatal aperture: confocal microscopy of PI stained leaf subsamples and cryo*-*scanning electron microscopy (cryo-SEM) of the frozen leaf subsamples (Van Gardingen et al., 1989). Cryo-SEM was chosen as the validation method because it was possible to examine and capture the effect of the signals generated during the early time points of tritrophic interactions in the context of the entire plant. *S.* Typhimurium leaf inoculation did not change or reduce the average stomatal aperture size at both 3 and 12 h post inoculation compared to the mock-inoculated control, referred to as mock from here forward (Figure 1A and Supplementary Figures S1A,B). Both confocal microscopy and cryo-SEM showed similar aperture profile under *S.* Typhimurium treatment. Using images obtained through the confocal imaging and cryo-SEM technique the stomatal width taken at the widest point was averaged for 40–60 stomata per leaf after which the average of all samples in a set were averaged (*n* = 3) (Figure 1A and Supplementary Figure S2). Treatment of both *S.* Typhimurium and *L. monocytogenes* showed stomatal aperture reduction at 6 h post inoculation, but only *L. monocytogenes* treatments induced a smaller stomatal phenotype at 12 h post inoculation compared to the control and *S.* Typhimurium treatment (Figure 1A and Supplementary Figure S1A). *S.* Typhimurium presented a phenotype like that of the mock after 3 h and was not different from the mock at 12 h post inoculation as well. Both the control and *S.* Typhimurium were significantly different (*p* < 0.05) from the reduced stomatal aperture observed in *L. monocytogenes* treated plants. A visual comparison shows that *L. monocytogenes* causes visibly smaller apertures while water treatments and *S.* Typhimurium treatments are visually similar (micrographs not shown). Besides altering the aperture sizes, *L. monocytogenes* leaf inoculation significantly increased the percentage of closed stomata on the abaxial leaf surface at both 3 and 6 h post inoculation compared to the control and *S.* Typhimurium treatments (Supplementary Figure S1B). Taken together, these data showed that leaf inoculation of *S.* Typhimurium can modulate stomatal aperture to keep stomates open post inoculation.

### *S.* Typhimurium Culture Filtrate (CFL) and Microbe-Associated Molecular Patterns (MAMPs) Modifies Stomatal Aperture in Lettuce

*Salmonella* Typhimurium treatments negated noticeable stomatal changes in lettuce plants. It is hypothesized that the cell-derived products from *S.* Typhimurium may be causing modulations in in plant innate responses. To facilitate this a filter sterilized culture filtrate (CFL) was prepared from *S.* Typhimurium, *sseB*, or *hilD* grown in a minimal media, these CFLs were used in a stomatal leaf assay. Each prepared CFL led to closure of stomates compared to the untreated control and *S.* Typhimurium post 3 h inoculation (Figure 1B). A heat-treated CFL prepared from *S.* Typhimurium showed a stomatal phenotype similar to live *S.* Typhimurium treated lettuce, suggesting that produced components from *S.* Typhimurium may trigger plant innate defense response to close stomates and these products were subsequently destroyed during a heating step (Figure 1B). Other known stomata modulating components such as lipopolysaccharides and flg22 were also evaluated for stomatal closure post 3 h inoculation. It is known that MAMPs and other cellular components derived from bacterial plant pathogens trigger plant innate defense response to close stomata (Melotto et al., 2006; Kumar et al., 2012). To test our results against purified *S.* Typhimurium specific LPS, a non-specific LPS (*P. aeruginosa*) and flg22 (10 μg mL^–1^) were applied to the stomatal leaf assay. Each of these cellular components caused stomatal closure statistically similar to *S.* Typhimurium CFL-challenged lettuce, except flg22 which caused 50% more closure than *S.* Typhimurium CFL (Figure 1B). The stomatal closure induced by these cellular components was 60 to 25% greater than the aperture means gathered in the control and live *S.* Typhimurium trials. The data suggests that *S.* Typhimurium but not the secreted CFL from *S.* Typhimurium can regulate stomatal apertures by suppressing the plant innate immune response of lettuce.

### *S.* Typhimurium Mutants Lacking Type-III Secretion System (T3SS) Virulence Factors Show Reduced Suppression of Stomatal Defense Response

**FIGURE 2:**
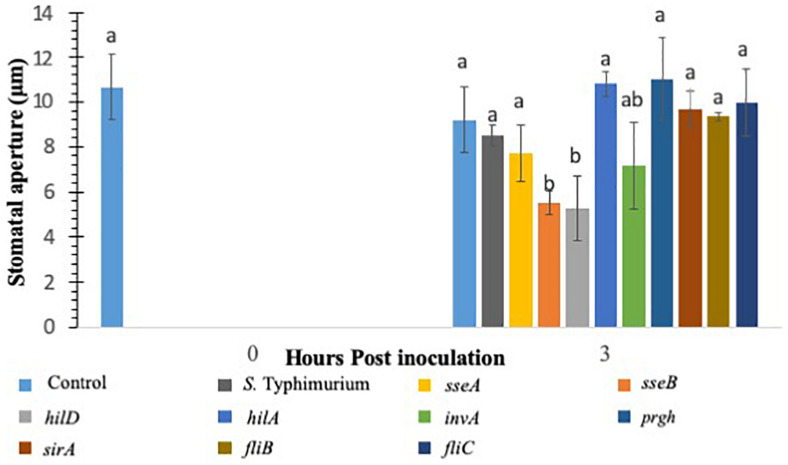
Stomatal closure in lettuce plants treated with *S.* Typhimurium and mutants of SPI1, SPI2, regulatory proteins controlling T3SS, Flagellin B, and Flagellin C. Aperture changes at 0 h remains unchanged, and 0 h represents only the water control. Different letters signify a difference at *p* < 0.05. For each time point the average was found from three plants with *n* = 40–60 stomata imaged per plant.

Various studies have shown that *S.* Typhimurium possesses components that are recognized in plants (Shirron and Yaron, 2011; Garcia and Hirt, 2014). The experiments involving CFL from *S.* Typhimurium showed that plants recognize secreted components from *S.* Typhimurium to launch a PAMP triggered immunity to close stomata. Furthermore, inoculation of *S.* Typhimurium serovars to *Arabidopsis thaliana* (Family: Brassicaceae) seedlings triggered MAPK activation and defense gene expression to a similar extent as that provoked by *P. syringae* inoculation (Schikora et al., 2008, 2011; Garcia and Hirt, 2014). We also showed that classical MAMPs such as flg22 and LPS induce PTI-stomatal closure in lettuce. We hypothesized that factors governed through the T3SS system in *S.* Typhimurium may modify stomatal defense response differently in lettuce. To this end, we used various T3SS mutants belonging to both SP1 and SP2 system in *S.* Typhimurium for modification of stomatal defense response in lettuce. Recently, *Salmonella* T3SSs and effectors were proposed to contribute to the plant colonization process (Garcia and Hirt, 2014). *S.* Typhimurium mutants in T3SS-1 and T3SS-2 induced stronger cell death and chlorosis symptoms and proliferated to lower levels in *Arabidopsis* leaves (Schikora et al., 2011). We tested all the *S.* Typhimurium 14028 mutants (*fljB*, *fliC*, *hilD*, *sseB*) by applying them to light adapted lettuce leaves and the stomatal aperture at 3 h post inoculation was recorded (Figure 2). Of the nine mutants applied to the stomatal assay only two resulted in consistent stomatal closure like that seen on plants treated with *S.* Typhimurium CFL (Figure 2). A majority of T3SS mutants caused no apparent stomatal closure at 3 h post inoculation. Most *Salmonella* serovars carry two flagellin-encoding genes, *fliC* and *fljB* (Silverman and Simon, 1980). The mutants lacking flagellar genes (*fljB* and *fliC*) in *S.* Typhimurium also showed lack of stomatal closure (Figure 2). It is shown that *S.* Typhimurium flagellin mutants triggered reduced defense responses in *Arabidopsis* and tomato (Iniguez et al., 2005). The T3SS mutants *hilD* and *sseB* caused stomatal closure that was significantly different (*p* < 0.05) then the mock or live *S.* Typhimurium (Figure 2). *hilD* is a transcription regulator for the master control of SPI1 and *sseB* lacks a functional T3SS in SPI2 (Ruiz-Albert et al., 2003; Ellermeier et al., 2005). Validation of *hilD* and *sseB* stomatal phenotype was completed using cryo-SEM; stomatal apertures of plants treated with *sseB* showed significant closure compared to *S.* Typhimurium at both 3 and 6 h post inoculation. *hilD-*challenged plants showed a significant change in stomatal aperture at only 6 h post inoculation (Supplementary Figures S2A,B). We also evaluated if host sensing by lettuce is mediated through a T3SS, to this end, the CFL from the two T3SS mutants (*hilD* and *sseB*) were added to lettuce leaves and compared with live *sseB* and *hilD* for stomatal apertures (Supplementary Figure S3). It should be noted that, both *hilD* and *sseB* challenged lettuce leaves led to greater stomatal closure compared to the WT. The CFL from both T3SS mutants led to stomatal closure as seen with WT CFL and live *sseB/hilD* cells, suggesting that the host sensing by lettuce is independent of T3SS and T3SS may be required to bypass stomatal defense in plants.

### Persistence and Internalization of *S.* Typhimurium Mutants Lacking Type-III Secretion System (T3SS) Virulence Factors and Flagellar Genes in Lettuce Grown Under Greenhouse Conditions

**FIGURE 3:**
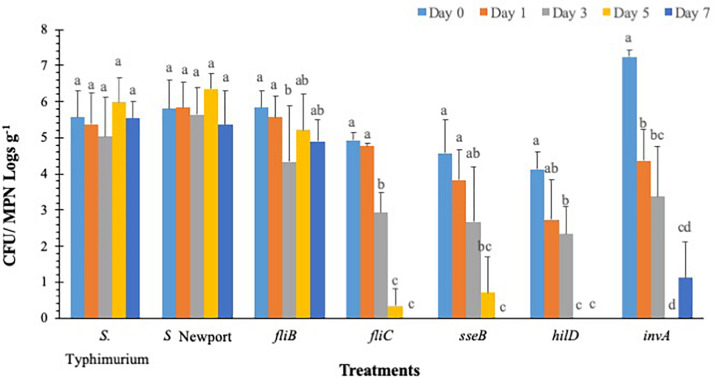
*S.* Typhimurium populations on lettuce over a 7-day period. Values represent the average of three replications. Standard deviation (±) for surviving *S.* Typhimurium population (Log_10_ CFU or MPN g^–1^) follows mean value. Letters *a, b, c* shows statistically significant differences (*p* < 0.05) between sampling days for the same strain.

*Salmonella* populations were significantly (*p* < 0.05) reduced in plants inoculated with T3SS (*hilD, sseB* and *invA*), and phase-1 flagellin (*fliC*) mutants by day 3 compared to wild type control (*S.* Typhimurium) except phase-2 flagellin (*fljB*) mutant (Figure 3). Mutants *hilD, sseB, invA*, and *fliC* populations were reduced between 2.3 and 3.3 MPN logs g^–1^ on day 3 compared to day 0 (4.1 and 7.2 log CFU g^–1^). *Salmonella* populations were further reduced to undetectable levels for *hilD invA, fliC*, and to 0.7 log MPN g^–1^ for *sseB* mutants, by day 5. The *fljB* mutants (4.3 to 5.8 CFU MPN logs g^–1^) and wild type control (5.0 to 6.0 logs CFU MPN logs g^–1^) showed similar survival throughout the 7-day trial. Similarly, in *S.* Newport inoculated plants, no significant (*p* < 0.05) change in pathogen populations (5.3–6.3 logs CFU MPNg^–1^) was observed on the lettuce surface, during the 7-day trial. Internalization assay results showed that *Salmonella* internalization was not detected in plants inoculated with *hilD* and *sseB* mutants, suggesting that these genes are vital for *Salmonella* internalization in lettuce (Supplementary Table S4). In *fliC* and *S.* Newport inoculated plants internalized *Salmonella* was observed until day 5 and in *invA* plants until day 1, of the trial. However, pathogen internalization was observed in *fljB* and wild type throughout the 7-day trial. It has been shown that *Salmonella* T3SS genes play different roles during plant-bacteria interactions based on plant species (Brandl et al., 2013). Iniguez et al. (2005) observed hyper-colonization of *Salmonella* T3SS SPI mutants than the wild type in alfalfa sprouts and *Arabidopsis thaliana*, On the contrary, in the current study, significantly low colonization was recorded in SPI mutants compared to the wild type. It is noteworthy that a previous study tested SPI- structural gene (s*ipB*) mutant, while the current study analyzed the effects of SPI-1 transcriptional regulator (*hilD*), SPI-2 (*sseB*), and SPI-1 (*invA*) mutants on survival. This could explain the differences in survival and internalization of mutants and suggests that these genes could affect *Salmonella* survival and persistence in lettuce leaves. Additionally, in the current study flagellar mutants showed similar internalization as wild type. This means that in the absence of either phase 1 or 2 flagellin genes, it could express flagella genes of the other phase (Olsen et al., 2012). This could explain similarities in survival and internalization pattern between flagellar gene mutants and wild type strain.

### Exogenous Hormones SA and ABA Induce Differing Stomatal Phenotypes in Lettuce Treated With *S.* Typhimurium

**FIGURE 4:**
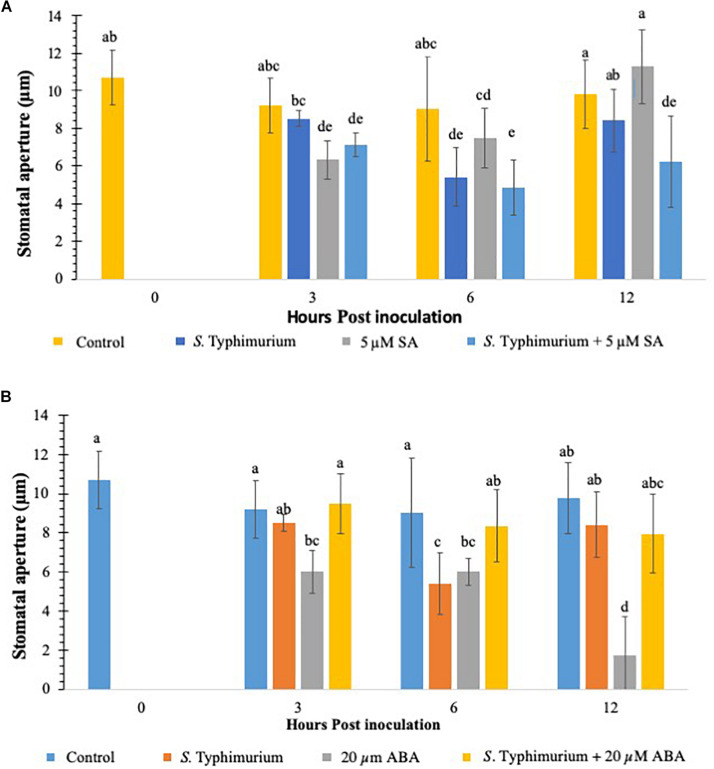
Stomatal aperture modulation in lettuce treated with *S.* Typhimurium co-inoculated with salicylic acid (SA) **(A)** or abscisic acid (ABA) **(B)**. Aperture changes at 0 h remains unchanged, and 0 h represents only the water control. Different letters signify a difference at *p* < 0.05. For each time point the average was found from three plants with *n* = 40–60 stomata imaged per plant.

Previous studies have shown that defense hormones play a critical role in the interaction between *S.* Typhimurium and plants (Garcia and Hirt, 2014.) Growth regulators such as SA, JA, and ET signaling pathways regulate *Salmonella* colonization in plants (Iniguez et al., 2005; Schikora et al., 2008). Studies have also shown that plants treated with *S.* Typhimurium biosynthesize SA and the induction of several marker genes of the SA pathway (Garcia et al., 2014). To this end, we evaluated if addition of exogenous SA and ABA, another key growth regulator which plays a critical role in stomatal physiology (Kumar et al., 2012) plays a role in co-inoculation with *Salmonella* in lettuce. Plants were inoculated with SA/ABA alone or co-inoculated with live *S.* Typhimurium and stomatal apertures were measured post 3–12 h post inoculation. When compared to other PTI induced plants like those treated with CFL and flg22, SA was similar in percentage and duration (Figure 4A). At 3 h post inoculation stomatal closure was apparent and different from the control treatment, and by 12 h post inoculation SA-challenged plants had returned to a normal stomata phenotype (Figure 4A). Exogenous SA in co-inoculation assays caused stomata to remain closed for longer than when just SA was applied. In fact, the stomatal stays closed twice as long (out to 12 hpi) when both SA and *S.* Typhimurium was present. The reasoning for this is that SA triggered a PTI that was not affected by *S.* Typhimurium’s phytopathogenic behavior therefore the presence of exogenous SA triggered PTI independently from *S.* Typhimurium. Despite these interesting observations, no hypersensitive response (patterned cell death) was observed on any plants, which may only be due to the 12 h length of the experiments. Patterned cell death was observed in research with *S.* Typhimurium applied to *Tabacum* and *Lycopersicum* (Family: Solanaceae) (Meng et al., 2013).

This study also evaluated the role of ABA in co-inoculation with *S.* Typhimurium in lettuce. It is shown that ABA plays a critical role in stomatal physiology under various biotic and abiotic conditions and is shown to be both synergistic and antagonistic with other plant hormones (Anderson et al., 2004; Merilo et al., 2015). When ABA was exogenously applied to the lettuce leaves, stomatal closure was observed just like on SA-challenged, plants (Figure 4B). Unlike SA, ABA caused closure at 3 h post inoculation and 6 h post inoculation but at 12 h post inoculation, when most plants in previous tests had reopened stomata, almost 100% of the stomata had closed (Figure 4B). The ABA induced closure significantly different (*p* < 0.01) from both SA and SA co-inoculated tests at 12 h post inoculation (Figures 4A,B). The co-inoculation with ABA and *S.* Typhimurium was set up by exogenously adding ABA (20 μM) and *S.* Typhimurium together. The stomatal apertures using confocal imaging were performed at 3, 6, and 12 h post inoculation. ABA co-inoculation with *S.* Typhimurium was performed with identical parameters and concentrations. The expected results with the co-inoculation of ABA with *S.* Typhimurium were to see stomatal closure from 3 until 12 h post inoculation as before; oddly enough the resulting stomatal phenotype was most similar to *S.* Typhimurium *-*challenged plants (Figures 1A,B). The data clearly showed that *S.* Typhimurium mediated stomatal opening was not actively closed by ABA treatment under co-inoculation, suggesting that *S.* Typhimurium may disrupt ABA biosynthesis and signaling *in planta* as a roundabout way of preventing stomatal closure. *SseB* and *hilD* were also tested in a co-inoculation experiment with ABA. At 3 h post inoculation, *sseB* showed stomatal closures similar to ABA treatment (Supplementary Figure S4). Co-inoculation of ABA with *sseB* led to stomatal reopening after 3 h post inoculation compared to *hilD* which at first had a phenotype more like that of *S.* Typhimurium but by 12 h post inoculation it had closed to that of the 20 μM ABA treated plants, a similar trend was observed with subsequent tests (Supplementary Figure S4). The later time point (6 and 12 h post inoculation) showed no reopening of stomates under a co-inoculation experiment with ABA and *hilD*, suggesting, the involvement of T3SS in suppressing ABA-mediated stomatal defense may occur early in plants interaction with *S.* Typhimurium.

To analyze if addition of ABA later to *S.* Typhimurium supplementation in lettuce leaves changes stomatal physiology, ABA was added 3 h post inoculation of *S.* Typhimurium supplementation. As shown previously, supplementation of ABA rapidly closes stomates in multiple plants (Kriedemann et al., 1972; Melotto et al., 2006). In comparison to co-inoculation experiment when ABA was added as a delayed application (3 h post inoculation), post *S.* Typhimurium treatment, stomatal re-opening with *S.* Typhimurium was observed (12 h post inoculation time point); though the reopening of stomates was not as prominent as that in the co-inoculation experiment (Supplementary Figure S5).

### *S.* Typhimurium Affects Genes in the ABA Biosynthesis and Translocation Pathway

**FIGURE 5:**
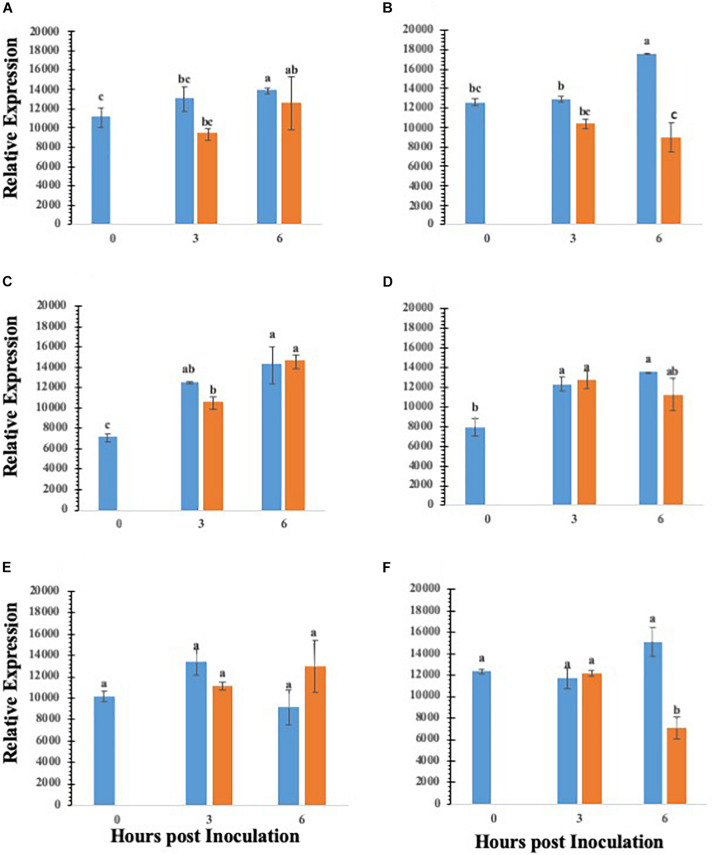
Gene expression analysis of various ABA biosynthesis genes in roots and leaves of lettuce plants treated with water (blue bar) or *S. enterica* Typhimurium (orange bar): *ABA3* leaf **(A)**; *ABA3* root **(B)**; *ZEP1* leaf **(C)**; *ZEP1* root **(D)**; *NCED3* leaf **(E)**; *NCED3* root **(F)**. Aperture changes at 0 h remains unchanged, and 0 h represents only the water control. Different letters signify a difference at *p* < 0.05.

Abscisic acid is a central regulator of stomatal closure (Acharya and Assmann, 2009), and our data showing that co-inoculation of ABA with *S.* Typhimurium led to reopening of stomates, we further analyzed the key ABA biosynthetic genes in lettuce treated with *S.* Typhimurium. The precise mechanism by *S.* Typhimurium for suppressing or blocking ABA effects on stomates remains to be shown. We analyzed transcript levels of ABA biosynthetic genes (*LsZEP1*, *LsNCED3* and *LsABA3*) in lettuce plants treated with *S.* Typhimurium. The data shows that transcript levels of ABA biosynthetic genes (*LsABA3* and *LsNCED3*) in roots and leaves of lettuce reduced post treatment with *S.* Typhimurium (Figures 5A–F). Treatment of *S.* Typhimurium to lettuce plants did not lead to changes in other transcript levels of genes from classical plant defense pathways (Supplementary Figure S6).

### *B. subtilis* UD1022 Colonization of *S*. Typhimurium Mutants Lacking Type-III Secretion System (T3SS) Virulence Factors and Flagellar Genes in Lettuce Grown Under Greenhouse Conditions

**FIGURE 6:**
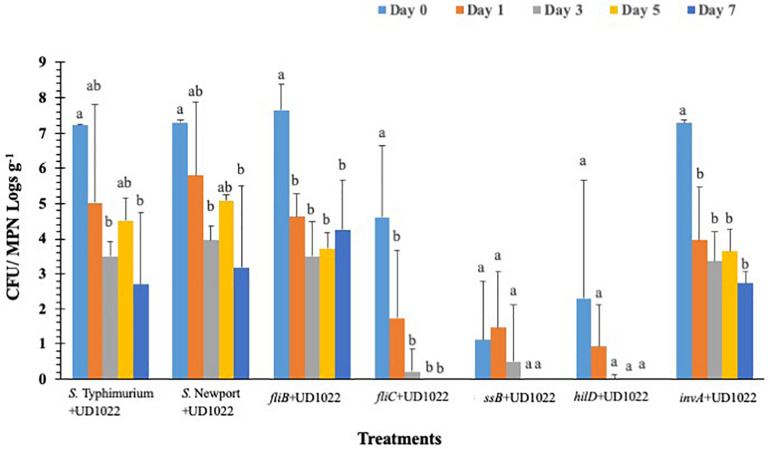
Surviving *S*. Typhimurium populations on lettuce surface with *B. subtilis* UD1022 under greenhouse conditions. Values represent the average of three replications. Standard deviation (±) for surviving *S*. Typhimurium population (Log_10_ CFU or MPN g^–1^) follows mean value. Letters *a, b, c* show statistically significant differences (*p* < 0.05) between sampling days for the same strain.

Overall *S*. Typhimurium populations were significantly (*p* < 0.05) reduced in all UD1022 treated groups by day 3 except *fljB* and *invA*, compared to the wild type (Figure 6). The *fljB* and *invA* showed significant (*p* < 0.05) decrease in pathogen survival on day 1 (4.6 and 4.0 logs CFU g^–1^, respectively) compared to day 0 (7.7 and 7.2 CFU g^–1^, respectively). Other mutant’s *fliC, hilD*, and *sseB* were reduced to undetectable levels in UD1022 treated plants compared to wild type strain (3.5 logs CFU g^–1^), by day 3. It is noteworthy that in *hilD*, and *sseB* groups had significantly (*p* < 0.05) lower (2.3-1.1 logs CFU g^–1^) levels of mutant’s colonization compared to the wild type (7.2 logs CFU g^–1^) strain, on day 0. Whereas the same mutants (*hilD*, and *sseB*) showed higher colonization on day 0 in the lettuce plants which were not treated with UD1022, suggesting an interaction between UD1022, these mutants and plant itself through a signal exchange to prevent bacterial attachment and internalization. In control groups, *S.* Typhimurium and *S.* Newport populations decreased significantly (*p* < 0.05) to 3.5 and 3.9 logs CFU g^–1^, respectively, by day 3, compared to day 0 (7.2 and 7.3 logs CFU g^–1^, respectively). *Salmonella* internalization was not detected in plants inoculated with mutants after UD1022 treatment, during the 7-day trial, except for *invA*, *S.* Typhimurium and *S.* Newport groups on day 0 (Supplementary Table S4).

## Discussion

It has been shown previously that few plant pathogens have an ability to modify stomatal physiology to ingress and cause infection (Melotto et al., 2006). Bacterial pathogens such as *P. syringae* DC3000 exploit a polyketide (coronatine) to reopen stomata in *Arabidopsis thaliana* to cause infection (Melotto et al., 2006; Kumar et al., 2012). On the contrary, plants get exposed to various human pathogens such as *P. aeruginosa* and *Enterococcus faecalis* and show classical signs of infection as seen previously with plant pathogens (He et al., 2004; Jha et al., 2005). Along the same lines, opportunistic human pathogens such as *E. coli* strains, *L. monocytogenes* and *Salmonella* serovars interact with plants and cause ingression and contamination (Gorski et al., 2009; Deering et al., 2012). The mechanisms by which opportunistic human pathogens, such as *Salmonella* spp., demonstrate plant ingression are not well understood. It is often speculated that opportunistic human pathogens such as *Salmonella* may use natural entry points (stomata) or mechanical injuries to ingress *in planta* (Melotto et al., 2006; Barak et al., 2011; Deering et al., 2012; Zheng et al., 2013). *Salmonella* can adapt well and is able to proliferate in some plant organs such as tomato fruit, more over there is overlap of plant pathogen and *Salmonella* mechanisms which shows the versatility and repurposing abilities of *Salmonella* (de Moraes et al., 2017). In this study, we report that *S.* Typhimurium 14028 may bypass the plant innate immune response to suppress stomatal defense for ingression. We also show that *S.* Typhimurium uses key T3SS factors to overcome stomatal defense for ingression. Rapid cryo-immobilization, cryo-SEM and confocal imaging were employed to capture the complex interaction between lettuce and *S.* Typhimurium in intact leaves with high accuracy and reproducibility. This approach revealed that plants treated with other opportunistic pathogens such as *L. monocytogenes* can induce stomatal closure, however, only the *S.* Typhimurium treatment showed ability to keep stomates open bypassing innate stomatal defense. We also showed that ability of *S.* Typhimurium to overcome stomatal defense was T3SS dependent. In addition, the SPI1 and SPI2 mutants of T3SS mutants revealed poor fitness and persistence under realistic growth conditions in lettuce. Using exogenous application of SA and ABA, we showed that *S.* Typhimurium may suppress ABA response in lettuce to keep the stomates open for longer durations. Using the gene expressions for ABA specific biosynthetic genes, we discovered that the addition of the *S.* Typhimurium, mediated stomatal non-closure mainly through a suppression of ABA biosynthetic pathway. This study suggests that pathogenic bacteria may associate with plants leading to development of key strategies by *Salmonella* to invade plants resulting in contamination.

### *S.* Typhimurium Live Cells and Cell Free Lysate Treatment Indicate the Critical Role of Stomatal Defense in Identifying Microbial-Derived Factors Regulating Innate Response

Previous studies showed that application of bacteria to isolated epidermal peels from *Arabidopsis thaliana* induced a MAMP-triggered immune response that closed stomata (Melotto et al., 2006; Zeng and He, 2010). Our data show that leaf inoculation of bacteria such as *L. monocytogenes* closed stomata at 3 h post inoculation; in contrast, *S.* Typhimurium treatments kept the stomata open until 12 h post inoculation. The rapid closure of stomata with *L. monocytogenes*, a classical innate defense response, suggests the response is most likely related to a MAMP-triggered immunity (Zeng and He, 2010). A similar response was observed when known MAMPs, such as LPS and flg22 were added to the lettuce leaves. Melotto et al. (2006) and Roy et al. (2013) showed that flg22 and LPS close stomata in the isolated leaf epidermal peels of Col-0 and lettuce plants, similarly, our data showed that supplementation of MAMPs such as LPS and flg22 closed stomata in lettuce. The stomatal closure by MAMPs was contrastingly opposite in case of *S.* Typhimurium treatment wherein no stomatal closure was observed post 3 and 12 h post inoculation. The data shown by Roy and Melotto (2019) showed treatment by *S*. Typhimurium SL1344 negated stomatal closure in Romaine at 4 h post inoculation under both 65 and 95% relative humidity. In contrast, cell free lysate (CFL) treatment to lettuce leaves from *S.* Typhimurium showed a classical MAMP based stomatal closure compared to live cell treatments, suggesting that cell free or secreted components from *S.* Typhimurium are recognized by plants to trigger innate response. The host sensing of cell free lysate from *S*. Typhimurium SL1344 was monitored and shown with human cells, wherein cell free lysates from *S.* Typhimurium SL1344 were recognized by the hosts leading to caspase-1-mediated proteolytic cell death contributing to pathogen clearance (Shivcharan et al., 2018). Interestingly, the heat-killed CFL treatment showed similar stomatal aperture as seen previously with live *S.* Typhimurium cells, suggesting that host sensing for innate stomatal defense requires secreted exo-metabolites. A recent study has shown that under high relative humidity conditions *S.* Typhimurium SL1344 but not *E. coli* O157:H7 suppresses stomatal defense response in Romaine compared to spinach, *E. coli* O157:H7 has also been shown to induce a higher degree of immune response from *Arabidopsis* and lettuce (Roy et al., 2013; Roy and Melotto, 2019). However, through the current study it is evident that host defense suppression and host sensing are two different layers of innate immune response that follow invasion of *S.* Typhimurium in lettuce.

### *S.* Typhimurium T3SS Components Regulate Both Host Sensing and Innate Stomatal Defense Response in Lettuce

To further test the hypothesis that *S.* Typhimurium can modulate the plant innate defense response several T3SS mutants of *S.* Typhimurium were tested with the stomatal assay. Our results showed the dependency of both SPI1 and the T3SS of SPI2 (*hilD* and *sseB*) in suppressing stomatal defense response at early time points of the exposure of *S.* Typhimurium. The mutants (*hilD* and *sseB*) are defective in SPI1, and the T3SS of SPI2, respectively. A lack of *hilD* lead to stomatal closure at time points consistent with that observed in *L. monocytogenes-*treated plants with confocal microscopy. *SseB* normally functions as a structural component of the SPI2 T3SS, and removal disrupted SPI2 functions (Ruiz-Albert et al., 2003). A strong stomatal closure was observed with lettuce leaves treated with *sseB* at 3 h post inoculation. The invasion protein mutant *invA* was able to partially suspend stomatal closure, resulting in a phenotype similar to both *S.* Typhimurium and *hilD, sseB* phenotypes.

The data shows that *S.* Typhimurium may need both SPI1 and SPI2 to fully subvert the basal immune response of *L. sativa* but may have more dependence on SPI2 rather than SPI1. Internalization assays showed that while *S.* Typhimurium was capable of internalizing and surviving on the plant surface concurrently: *fliC*, *hilD* and *sseB* did not persist on the leaf and no internalized cells were isolated within the 7-day long trial. As seen in stomatal assays *invA* was slightly better able to survive, on the surface and was isolated from the apoplast when *hilD* and *sseB* were not recovered at any time point from inside leaf samples. We concluded that *invA* lacking an essential invasion protein was partially competent as a plant pathogen and that InvA is a possible effector responsible for plant immune suppression.

Intercellular transportation and colonization by *Salmonella* is a pathogenic trait that allows rapid spread and infection in mammalian hosts and is mediated by SPI2 (Löber et al., 2006). Previous work has shown that *Salmonella* ingress in plant tissues using natural openings as stomata, trichomes or injured roots (Barak et al., 2002, 2011; Deering et al., 2012). Our studies and that of others showed *S.* Typhimurium T3SS mutants have high morbidity rates on and in plants, suggesting that both SPI-1- and SPI-2-encoded apparatuses are necessary to establish robust proliferation and ingression in plants (Schikora et al., 2011). Using a brush inoculation technique, we did not observe any hypersensitive response or lesion formation with both *S.* Typhimurium and T3SS mutants in plants. Schikora et al. (2011), showed a classical HR and lesion formation followed with chlorosis using *S.* Typhimurium and T3SS mutants by using blunt injection technique. It should be mentioned that a blunt injection technique may bypass both leaf and stomatal defense response in plants. A similar study used tobacco as a model system and showed that *S.* Typhimurium, but not the T3SS mutant *invA*^–^, were able to suppress the oxidative burst and the increase of extracellular pH after inoculation, suggesting that *Salmonella* actively suppresses plant defense mechanisms using the SPI-1 encoded T3SS (Shirron and Yaron, 2011). Chalupowicz et al. (2017) found no evidence that *Salmonella* can translocate effectors from plant pathogenic origin, and no HR response was generated by *Salmonella*. It does appear that *Salmonella* can influence plant physiology but is dependent on experimental protocols and does not always follow previously published doctrine.

Our data showed that treatment with *S.* Typhimurium may keep the stomates open for possible ingression, similarly, Roy et al. (2013), revealed that *S.* Typhimurium treatment of *Arabidopsis* and lettuce leaves triggered reduced stomatal closure as compared with *E. coli* (Roy et al., 2013). Similar to the results presented here, *Salmonella* treated leaves showed stronger stomatal reopening 4 h after bacterial inoculation (Roy et al., 2013; Roy and Melotto, 2019). Our studies show that *Salmonella* may keep the stomates open for longer duration and the suppression of stomatal defense is partly dependent on SPI1 triggered T3SS during the early onset of *Salmonella* exposure with lettuce plants.

### ABA Has a Central Role in *Salmonella’s* Suppression of Stomatal Defense for Ingression

Stomatal regulation and function are affected by abiotic, biotic and hormonal interactions, and typically, ABA plays an overriding role during stomatal closure (Acharya and Assmann, 2009). Previously, it was shown that beneficial soil microbes may mediate stomatal closure mediated through ABA (Kumar et al., 2012). Suppression of ABA biosynthetic pathway was critical for *Salmonella* mediated suppression of stomatal defense. Supplementation with ABA in a co-inoculation experiment showed that *S.* Typhimurium may override ABA’s effect to close stomates. In addition, *S.* Typhimurium inoculated plants showed the downregulation of transcript levels of ABA biosynthetic genes. Specifically, biosynthetic genes such as LsNCED3, and LsABA3 were all downregulated post *S.* Typhimurium treatment at 6 h post inoculation. Both LsZEP1 and LsNCED3 are involved in early steps of ABA biosynthesis (Xiong and Zhu, 2003). LsZEP1 regulates the conversion of zeaxanthin to neoxanthin and is reported to be regulated by circadian rhythm (Taylor et al., 2000) in contrast, the regulation of LsZEP in roots is regulated under drought conditions (Taylor et al., 2000). Similarly, the expression of LsNCED3 is highly regulated under both biotic and abiotic stress regimes (Xiong and Zhu, 2003). It is also shown that the expression of LsNCED3 was early under abiotic/biotic stress regime (Qin and Zeevaart, 1999). The *S.* Typhimurium treatment to leaves of lettuce led to downregulation of LsNCED3, but not LsZEP1, post 3 and 6 h post inoculation in roots suggesting the inducible nature of some ABA biosynthetic genes, previous work has not used lettuce as a model organism when observing ABA expression following a biotic stressor. Frey et al. (2012), remarks that a slight change in ABA levels due to mutation of NCED3 can lead to reduced vegetative growth and that other NCED genes only partially contribute to ABA production if NCED3 is removed. Our data showed that *S.* Typhimurium reduced expression of NCED3 in both the roots and leaves, suggesting that both ABA biosynthesis and translocation can be modulated by *S.* Typhimurium treatment causing a change in key ABA biosynthesis genes. The ABA modulation by *S.* Typhimurium was independent of T3SS apparatus. Study with an ABA overproducer transgenic line in tomato (Sp5) showed that the decline rate of *Salmonella* on the leaf surface of Sp5 was significantly higher than that of its wild type “Ailsa Craig” (Gu et al., 2013). In contrast, there was no significant difference for the internal decline rate of Salmonella in between Sp5 and the parental line (Gu et al., 2013). How *S.* Typhimurium modulates temporal production and translocation of ABA *in planta* needs to be elucidated. There is a tempting possibility that ABA directly may impact the growth of *Salmonella in planta*, which needs to be evaluated.

### *B. subtilis* UD1022 Prevents *S.* Typhimurium Ingression and Persistence

It was previously shown that *B. subtilis* UD1022 promotes biomass, drought tolerance, and pathogen resistance in many plant species (Kumar et al., 2012; Bais et al., 2014; Zheng et al., 2018). Recently, it was demonstrated that the addition of the rhizobacteria, *B. subtilis* UD1022 to the roots of the *A. thaliana* plants restricted the entry of the foliar pathogen *Pst*DC3000 through the stomata (Kumar et al., 2012). The root-inoculation apparently triggered an *in planta* signal that resulted in the closure of the guard cells, which was more pronounced in the presence of the foliar pathogen *Pst*DC3000 (Kumar et al., 2012). Both abscisic acid (ABA) and SA were shown to be involved in the early closure of the stomata thus minimizing the pathogen entry points on the leaf surface (Kumar et al., 2012). The influence on the stomatal closure was observed as a general phenomenon with the entire *Bacillus* species tested, which indicates that members of this genus can modulate the stomatal phenotype. A recent study involving *B. amyloliquefaciens* FZB42 showed stomatal closure and protection in *Nicotiana benthamiana* caused by *Phytophthora nicotianae* (Wu et al., 2018). Both the studies (Kumar et al., 2012 and Wu et al., 2018) *per se* portrays the involvement of the primary signaling components, mainly SA and ABA, during the beneficial interaction of *Bacillus* spp. with the plant root and its effect on the stomatal behavior.

In this article UD1022 was utilized to prevent a foodborne pathogen from both internalizing and setting up a persisting population within lettuce. We proved that UD1022 after associating with lettuce for at least 48 h induced no adverse stress which was also observed in Kumar et al. (2012) but on *A. thaliana*. UD1022 primed lettuce showed a quick response to *S.* Typhimurium in that stomata quickly closed and remained closed for at least 12 h, while water treated plants did not exhibit a synonymous response. By observing successfully closed stomata it was determined that UD1022 had effectively induced system resistance (ISR), and work could continue to examine the actual amount of ingression, if any, and persistence of *S.* Typhimurium on UD1022 inoculated plants. Initial persistence assays showed that *S.* Typhimurium and *S.* Newport is very capable at getting into and surviving in the plant as well as surviving on the surface while T3SS mutants were not. The same was not observed on UD1022 primed plants, where *S.* Typhimurium maintained a small external population but did not internalize or perhaps survive inside the plant long enough to be detected. We hypothesized that UD1022 effectively induces ISR, which returns defense to immune limited plants, in this case lettuce. We do know that UD1022 is not outrightly toxic to *Salmonella* as shown in Markland et al. (2015). It is probable that an increase in defense related genes such as PR-1 leads to induction of ROS generation which is both a method for raising the alarm in plants and can be antagonistic to many bacteria. Evidence that UD1022 provides defense against foodborne pathogen contamination has been proven in the lab but is yet be shown in a field grown setting, although the methods used in the paper do illustrate a potential use in hydroponic systems which needs be evaluated with a more complex production system. Studies have shown that rhizobacteria could act a biocontrol against plant pathogen through enhanced plant defense response (Srivastava et al., 2016; Backer et al., 2018). A study showed that plant growth promoting rhizobacteria *B. amyloliquefaciens* (SN13) can effectively control a pathogenic plant fungus i.e., *Rhizoctonia solani in vitro* and in rice (Srivastava et al., 2016). Authors suggested that the rhizobacteria can elicit immune response in plant by modulating metabolic and biochemical pathways, which could play an important role in protecting plant against infection and pathogens. Similarly, in the current study, plants primed/inoculated with UD1022 displayed decreased internalization incidence in *Salmonella* mutants and controls, suggesting that rhizobacteria could be of importance to plant against human pathogen. Moreover, low levels of bacterial colonization observed in SPI mutants (*hilD*, and *sseB*) inoculated plants along with UD1022 indicates that these genes may play a critical role to override signal transmission in plant against *Salmonella*. In the absence of these genes, the plant growth promoting rhizobacteria UD1022 was able to express signals and plant immune response with decreased internalization and surface colonization. The peculiar response in colonization observed in *invA* further supports the notion that *invA* is involved in immune suppression but does not act alone, and UD1022’s ISR is able to overcome these effects through multiple pathways. A study by Hsu and Micallef (2017) showed a similar result, where the plant growth promoting rhizobacteria i.e., *Pseudomonas* strains S2 and S4 significantly reduced *S.* Newport population on spinach, lettuce and tomato surface. The authors evaluated the wild type strain and suggested that increased leaf nitrogen content might have limited pathogen survival on the surface. Whereas, the current study revealed the effect of *S.* Typhimurium virulence genes and their interactions with rhizobacteria on its survival and persistence on lettuce surface. Furthermore, UD1022 treatments caused only transient stomatal closure which corrects by 48 h post inoculation to stomatal apertures observed on control plants (Supplementary Figure S7). Little change in stomatal conductance was observed on dark adapted or light adapted UD1022 treated plants compared to control plants (Supplementary Figure S7). The photosynthetic efficiency of UD1022 treated plants compared to those left untreated was not perturbed over a 60 h time series (data not shown). The data related to transpiration and stomatal conductance showed that application of UD1022 may not affect plants physiologically and may act as an effective biocontrol mechanism against foodborne pathogens.

## Concluding Remarks

Taken together, these observations suggest that *S.* Typhimurium uses a T3SS-delivered effector protein to suppress the immune stomatal defense system. The two-tier system of stomatal innate defense response and host sensing was shown using a CFL from *S.* Typhimurium. The study suggests that exo-metabolites from *Salmonella* may be sensed by plants to trigger stomatal defense response. How *Salmonella* achieves the delivery of effectors across plant cell walls and plant plasma membranes remains unclear. However, numerous phytopathogenic bacteria (like *Pseudomonas*, *Erwinia* and *Xanthomonas* spp.) are known to utilize T3SS to deliver effector proteins across plant cell wall, indicating that the plant cell wall is not a sufficient barrier to prevent bacteria from effector delivery (Schikora et al., 2011). *Salmonella* remains an important foodborne pathogen and food safety hazard for both pre- and post-harvest conditions. Knowledge pertaining to non-host survival in the external environment is useful in development of novel strategies to mitigate risks of contamination. The use of PGPRs may elucidate a viable and safe method to affect plant-pathogen interactions.

## Data Availability Statement

The dataset is available and uploaded through dryad. NJ, HB, KK, PL (2020), Evasion of plant innate defense response by *Salmonella* on Lettuce, v2, Dryad, Dataset, https://doi.org/10.5061/dryad.bg79cnp76.

## Author Contributions

All authors designed the experiments, edited and contributed to the final manuscript, and approved for submission. NJ completed lab work pertaining to microscopy and genetic expression. PL designed and completed persistence, internalization assays and *Bacillus subtills* efficacy trials.

## Conflict of Interest

The authors declare that the research was conducted in the absence of any commercial or financial relationships that could be construed as a potential conflict of interest.

## Abbreviations

ABA: abscisic acid
CFL: culture filtrate
CFU: colony forming units
h post inoculation or hpi: hours post inoculation
LPS: lipopolysaccharide
SA: salicylic acid
SPI1, SPI2: salmonella pathogenesis islands One or Two
T3SS: type three secretion system.
