# Corrigendum: Evasion of Plant Innate Defense Response by *Salmonella* on Lettuce

**DOI:** 10.3389/fmicb.2022.875831

**Published:** 2022-05-30

**Authors:** Nicholas Johnson, Pushpinder K. Litt, Kalmia E. Kniel, Harsh Bais

**Affiliations:** ^1^Department of Plant and Soil Sciences, University of Delaware, Newark, DE, United States; ^2^Delaware Biotechnology Institute, University of Delaware, Newark, DE, United States; ^3^Department of Animal and Food Sciences, University of Delaware, Newark, DE, United States

**Keywords:** *S. enterica*, *L. monocytogenes*, T3SS, stomata, innate defense response, food safety, pattern triggered immunity, hormonal response

In the original article, there was an error. The method section in our manuscript for describing statistical analysis was not adequately worded to reflect the statistical analysis performed for the data set. The following describes the statistical analysis performed with the data sets. A correction has been made to **Materials and Methods, Statistical Analysis**:

“All the experiments were repeated in triplicate per treatment. Bacterial populations, recovered at each sampling point, were converted to log10 CFU/g or MPN/g and mean values of the three replicates obtained. The limit of detection was 1 log CFU/g. Stomatal width at each time point was captured with confocal microscopy and measured using ImageJ. Data were analyzed using a one-way analysis of variance to determine the effect of treatments, student's t-test was performed to compare the means of bacterial populations, stomatal width, and relative gene expression over time by using JMP (JMP v.14 software; SAS Institute Inc., Cary, NC) at a significance level of *P* < 0.05.”

In addition, the Flagellin mutant (fljB) used in the study is incorrectly referred as fliB. This has been corrected throughout.

The authors apologize for these errors and state that this does not change the scientific conclusions of the article in any way. The original article has been updated.

## Publisher's Note

All claims expressed in this article are solely those of the authors and do not necessarily represent those of their affiliated organizations, or those of the publisher, the editors and the reviewers. Any product that may be evaluated in this article, or claim that may be made by its manufacturer, is not guaranteed or endorsed by the publisher.

