# Supplementary material for: Evasion of Plant Innate Defense Response by Salmonella on Lettuce
Source: Front Microbiol. 2020 Apr 3;11:500. doi: 10.3389/fmicb.2020.00500 (PMC7147383; doi:10.3389/fmicb.2020.00500)
Supplement: Supplementary file 2 [file Data_Sheet_2.docx]

Supplementary Material

**Supplementary Table 1.** Strains used in the study.

| **Strains** | **Description** | **Antibiotic** | **Source:** |
| --- | --- | --- | --- |
| *S.* Typhimurium 14028s | Wild type | None | C. Altier |
| *S.* Typhimurium *hilD* | *Salmonella* Pathogenicity Island-1 (SPI-1) transcriptional regulator hilD mutant | Chloramphenicol | C. Altier |
| *S.* Typhimurium  *hilA* | *Salmonella* Pathogenicity Island-1 (SPI-1) transcriptional regulator hilA mutant | Kanamycin | C. Altier |
| *S.* Typhimurium *sseB* | T3SS mutant ( SPI2) sseB Chaperone protein mutant | Tetracycline | C. Altier |
| *S.* Typhimurium *sseA* | T3SS mutant ( SPI2) | Tetracycline | C. Altier |
| *S.* Typhimurium *fliC* | Phase 1 flagellin gene *fliC* mutant | Chloramphenicol | C. Altier |
| *S.* Typhimurium *fljB* | Phage 2 flagellin gene *fliB* mutant | Kanamycin | C. Altier |
| *S.* Typhimurium *invA* | SPI-1 regulatory mutant | Kanamycin | C. Altier |
| *S.* Typhimurium  *prgh* | SPI-1 T3SS effector mutant | Tetracycline | C. Altier |
| *S.* Typhimurium  *sirA* | Global SPI regulatory mutant | Tetracycline | C. Altier |
| *S.*  Newport | Wild type | Rifampicin | K. Kniel |
| *L. monocytogenes­*-GFP | Nonmutant- GFP tagged | Erythromycin | L. Gorski |
| *B. subtilis UD1022* | Wild Type | No Selection | H. Bais |

| Genes | Hotstart (95^o^C) | Annealing | Binding |  | Cycles | Curing |
| --- | --- | --- | --- | --- | --- | --- |
| ACTIN | 5 minutes | 94^o^C/ 15 s | 54^o^C/ 30s | 72^o^C/ 30 s | 40 | 72^o^C/ 5 min |
| PR1 | 5 Minutes | 94^o^C/ 15 s | 57^o^C/ 30s | 72^o^C/ 30 s | 35 | 72^o^C/ 5 min |
| NPR1 | 5 Minutes | 94^o^C/ 15 s | 53.5^o^C/ 30 | 72^o^C/ 30 s | 40 | 72^o^C/ 5 min |
| PDF1.2 | 5 Minutes | 94^o^C/ 15 s | 54.5^o^C/ 30s | 72^o^C/ 30 s | 40 | 72^o^C/ 5 min |
| NCED1 | 5 Minutes | 94^o^C/ 30 s | 55^o^C/ 30s | 72^o^C/ 30s | 40 | 72^o^C/ 5 min |
| NCED2 | 5 minutes | 94^o^C/ 15 s | 57^o^C/ 30s | 72^o^C/ 30 s | 30 | 72^o^C/ 5 min |
| **Supplementary Table 2.** Rt-PCR conditions to evaluate ABA biosynthesis gene expressions in lettuce post treatment with *S. enterica* Typhimurium **(B)***.*   \| Genes \| Hotstart (95^o^C) \| Annealing \| Binding \|  \| Cycles \| Curing \| \| --- \| --- \| --- \| --- \| --- \| --- \| --- \| \| ABA3 \| 5 minutes \| 94^o^C/ 15 s \| 56^o^C/ 30s \| 72^o^C/ 30 s \| 40 \| 72^o^C/ 5 min \| \| NCED3 \| 5 Minutes \| 94^o^C/ 15 s \| 57^o^C/ 30s \| 72^o^C/ 30 s \| 35 \| 72^o^C/ 5 min \| \| ZEP1 \| 5 Minutes \| 94^o^C/ 15 s \| 57^o^C/ 30 \| 72^o^C/ 30 s \| 40 \| 72^o^C/ 5 min \| \| ABA3 \| 3 minutes \| 94^o^C/ 30 s \| 55.2^o^C/ 30s \| 72^o^C/ 30 s \| 35 \| absent \| \| NCED3 \| 3 Minutes \| 94^o^C/ 30 s \| 57^o^C/ 30s \| 72^o^C/ 30 s \| 35 \| absent \| | | | | | | |
|  |  |  |  |  |  |  |

|  |
| --- |
|  |
|  |
|  |
|  |
|  |
|  |
|  |
|  |

**Supplementary Table 2.** Rt-PCR conditions to evaluate classical plant defense gene expressions in lettuce post treatment with *S. enterica* Typhimurium **(A)***.*

**Supplementary Table 3.** Details of the primers used in the study.

|  | **Oligo-DNA Primers** | |  |
| --- | --- | --- | --- |
| **Gene** | **Forward Primer** | **Reverse Primer** | **Reference** |
| ACTIN | CAAGCCGTTCTTTCCCTGTA | TCCTGCTGAGGTCGTGAATG | (Markland et al. 2017) |
| PR1 | GAAGGGTTGGGTGTGCTAGA | CACAAGAAACAAGGGCGTAG | (Markland et al. 2017) |
| NPR1 | TCGATCGTCTATCGGAAACC | TCACATTGCGATTCTTGTCC | (Markland et al. 2017) |
| PDF1.2 | GCCATCTTCTCTGCTTTTGAA | ACACAAGACACTGCGACGAC | (Markland et al. 2017) |
| NCED2 | TCGACTCTTTGTGCACTTCATACTC | CAACGGGACAAGTTGAGGTTTTA | (Huo et al. 2013) |
| NCED1 | CGCAATCACCGAGAACTTTGT | GGCGATCCTCCTTTTATCATTTC | (Huo et al. 2013) |
| NCED3 | AGCTCAGCTTGGTTCCCTGTTATA | CTTCACAAACTGGCTGAAAACGTAT | (Huo et al. 2013) |
| ABA3 | CAGCTCTAGCTTGACCTCATCA | CCAAGGACACTGTTGTGGTTCT | (Argyris et al. 2008 ) |
| ZEP1 | GGGCTACACTTGTTACACTGGGATA | GCCCCAAGAACACCCGATA | (Huo et al. 2013) |

**Supplementary Table 4.** Detection of internalized *S.* Typhimurium WT, *S.* Newport, Flagellin (*fli*) and T3SS mutants in lettuce leaves over 7-day period, with or without *Bacillus subtilis* UD1022. +/- indicates presence or absence of the strains in planta.

| **Treatments** | **Day 0** | **Day 1** | **Day 3** | **Day 5** | **Day 7** |
| --- | --- | --- | --- | --- | --- |
| *S.* Typhimurium WT only | **+** | **+** | **+** | **+** | **+** |
| *S.* Typhimurium WT + UD1022 | **+** | **-** | **-** | **-** | **-** |
| *S*. Newport only | **+** | **+** | **+** | **+** | **-** |
| *S*. Newport + UD1022 | **+** | **-** | **-** | **-** | **-** |
| *fliB* only | **+** | **+** | **+** | **+** | **+** |
| *fliB* + UD1022 | **-** | **-** | **-** | **-** | **-** |
| *fliC* only | **+** | **+** | **+** | **+** | **-** |
| *fliC* + UD1022 | **-** | **-** | **-** | **-** | **-** |
| *sseB* only | **-** | **-** | **-** | **-** | **-** |
| *sseB* + UD1022 | **-** | **-** | **-** | **-** | **-** |
| *hilD* only | **-** | **-** | **-** | **-** | **-** |
| *hilD* + UD1022 | **-** | **-** | **-** | **-** | **-** |
| *invA* only | **+** | **+** | **-** | **-** | **-** |
| *invA* + UD1022 | **+** | **-** | **-** | **-** | **-** |

**Legends to Supplementary Figures.**

**Supplementary Figure 1.** A schematic that shows the area of the adaxial leaf surface used for both confocal and cryo-SEM and the method adopted for measuring stomatal aperture sizes using Image J software. Three leaves were picked for stomatal measurements and 40-60 stomates were monitored for stomatal aperture measurements from each leaf. Scale bar=30 microns (**A)**. Percentage of number of closed stomata post *S. enterica* Typhimurium and *L. monocytogenes* leaf inoculation. Stomata were counted using images obtained using cryo-SEM or confocal randomly at 3 and 6h. **=(p<0.05) (**B**).

**Supplementary Figure 2.** Lettuce leaves treated with *S.* Typhimurium, *sseB*, or *hilD* were sub sampled and coated in a freezing medium and sputter coated with gold-palladium before cryo-SEM at 3 and 6-h post-inoculations (**A)**. The leaves were imaged at 500x with 40-60 stomata images per leaf, 3 leaves were imaged at each time point. Representative electron-micrographs at 500X, stomata aperture reduction noticeable with *sseB* and *hilD* compared to control and *S.* Typhimurium treatments (**B)**. **=(p<0.05). Aperture changes at 0 hour remains unchanged, and 0 hour represents only the water control.

**Supplementary Figure 3.** Comparison of innate immune response in terms of stomatal closure by *S.* Typhimurium and T3SS mutants on lettuce, different letters signify a difference at p<0.01 (the mean is an average 3 leaves with 40-60 stomata counted per leaf). CFL in the panel refers to cell free culture filtrate. ***= p<0.001. Aperture changes at 0 hour remains unchanged, and 0 hour represents only the water control.

**Supplementary Figure 4.** Stomatal aperture modulation in lettuce treated with *S.* Typhimurium T3SS mutants (*sseB* and *hilD*) co-inoculated with abscisic acid (ABA). Stomatal apertures were measured 3, 6 and 12 h post inoculation. Different letters signify a difference at p<0.05. The mean is an average 3 leaves with 40-60 stomata counted per leaf. Aperture changes at 0 hour remains unchanged, and 0 hour represents only the water control.

**Supplementary Figure 5.** Stomatal aperture modulation in lettuce plants pre-treated with *S.* Typhimurium with delayed administration of ABA (3 h of post inoculation of *S.* Typhimurium). Stomatal apertures were measured 3, 6 and 12 h post inoculation. The mean is an average 3 leaves with 40-60 stomata counted per leaf. Aperture changes at 0 hour remains unchanged, and 0 hour represents only the water control. Different letters signify a difference at p<0.05.

**Supplementary Figure 6.** Gene expression analysis of classical plant defense and ABA biosynthetic genes [PR1 **(A)**; NPR1 **(B)**; PDF1.2 **(C)**; NCED2 **(D)**, NCED1 **(E)**] in lettuce treated with *S.* Typhimurium and TT3S mutants. Lettuce plants were leaf inoculated with S. Typhimurium wild type and T3SS mutants. ns refers to no significance.

**Supplementary Figure 7.** *B. subtilis* UD1022-mediated stomatal closure in lettuce is transient. The panel depicts the average stomatal closure and the relative transpiration at the same times from 0 to 60 h post inoculations. Initial UD1022 closure was observed from 4-8 h post inoculation. No significant changes in the transpiration rates were observed in between the untreated and UD1022 treated lettuce plants. The shaded portions in the panel refers to the dark phase in the circadian rhythm.
